# A Public Health Emergency of International Concern? Response to a Proposal to Apply the International Health Regulations to Antimicrobial Resistance

**DOI:** 10.1371/journal.pmed.1001021

**Published:** 2011-04-19

**Authors:** Adam Kamradt-Scott

**Affiliations:** Department of Global Health & Development, London School of Hygiene & Tropical Medicine, London, United Kingdom

## Abstract

Adam Kamradt-Scott critiques a proposal to apply the International Health Regulations (IHR) to the global health threat of antimicrobial resistance.

**Linked Policy Forum**

This Perspective discusses the following new Policy Forum published in *PLoS Medicine*:

Wernli D, Haustein T, Conly J, Carmeli Y, Kickbusch I, et al. (2011) A Call for Action: The Application of the International Health Regulations to the Global Threat of Antimicrobial Resistance. PLoS Med 8(4): e1001022. doi:10.1371/journal.pmed.1001022 http://www.plosmedicine.org/article/info:doi/10.1371/journal.pmed.1001022

Stephen Harbarth and colleagues argue that the International Health Regulations (IHR) should be applied to the global health threat of antimicrobial resistance.

There is little question that the international spread of antimicrobial resistance (AMR) is an alarming development that the global public health community must confront as a matter of some priority. Reducing the incidence of new cases will take the concerted effort and participation of all community sectors, and will require strong political leadership. The World Health Organization (WHO) is best placed to lead the international community's efforts to tackle this phenomenon, and as evidenced by the release of a new fact sheet on 21 February 2011 [1], the WHO Secretariat is acutely aware of the dangers microbial resistance presents.

In the paper “A Call for Action: The Application of the International Health Regulations to the Global Threat of Antimicrobial Resistance” published this week in *PLoS Medicine* [2], Stephen Harbarth and colleagues make the case to utilize the revised International Health Regulations (2005) (IHR 2005) to encourage Member States to report all cases of AMR as they fulfil “at least two” of the criteria for a public health emergency of international concern (PHEIC). While I commend the authors for seeking to further the field of understanding relating to the interpretation, application, and implementation of the IHR, I disagree with their proposal. For reasons of brevity, I will outline just three reasons here.

## The Object and Purpose of the IHR

Firstly, the fact that no international agreement to address AMR currently exists is insufficient justification for co-opting the IHR. This is principally because the IHR were never intended as a blanket framework to tackle all disease threats. When they were first created in 1951, the purpose of the IHR was described as “to ensure the maximum security against the international spread of diseases with a minimum interference with world traffic” [3]. Despite the multiple disease threats in the 1950s, only six were considered serious enough to warrant a new international agreement. Importantly, this was not only because of the clear ability of these six diseases to cause widespread human suffering, but also their ability to disrupt international trade. In the 2005 revisions, the scope of the IHR was expanded by adopting a broader definition of “disease” [4], but their essential function remained unchanged—they are a framework to guide inter-state behaviour when confronted with an acute public health emergency that threatens to disrupt international trade and travel.

To emphasise this dual focus, the revised IHR introduced the concept of a PHEIC. As defined in Article 1, a PHEIC is an “extraordinary event” that constitutes a public health risk, which may also require a coordinated international response. While outbreaks of carbapenem-resistant Enterobacteriaceae (CRE) are a worrying development that may warrant reporting under the IHR (and provision is already made for this within Annex 2), it is hard to appreciate that the global spread of AMR counts as “extraordinary” given that resistance has been a regular phenomenon since the invention of antibiotics in the 1940s and multidrug AMR has been anticipated for at least the past three decades. Moreover, the definition of “public health risk” emphasises “a serious and direct danger” [4]. A sense of immediacy can be appropriately inferred from this qualification—immediacy further corroborated by the inclusion of the term “emergency” within PHEIC—supporting the commonly held view that the IHR are designed to deal with acute (as opposed to chronic) public health conditions that are readily transmissible and disruptive to international trade.

## Pragmatism

A second reason is pragmatism. Put simply, the amount of information generated by mandatory reporting of every AMR case (irrespective of whether they constitute a PHEIC or not) would likely overwhelm not only Member States, but also the WHO Secretariat. It should be recalled, for instance, that the syndromic reporting trial that was initially trialled in the late 1990s to determine the suitability of syndromic reporting systems for the revised IHR framework had to be abandoned prematurely, largely because of the overwhelming number of reports it generated [5]. The WHO Secretariat simply did not have the resources to deal with the amount of information the trial produced. Given that the WHO is already facing significant budgetary constraints heading into the next financial period [6], available resources have to be a consideration in assessing the Harbarth proposal, as does the technical capacity of Member States and their willingness to report cases of AMR under the IHR. Indeed, it is critical at this juncture when many low-income countries are already struggling to strengthen and maintain disease surveillance capacities to meet their obligations under the IHR, not to overburden them further by requiring National IHR Focal Points to report each and every disease cluster. Unless a specific outbreak of AMR cases poses an imminent risk to global public health and fulfils the criteria outlined in Annex 2 of the revised IHR, other mechanisms can and should be used to report such occurrences. Broadening the scope beyond this is both inappropriate and impractical.

## The IHR Are Only a Framework

In their paper, Harbath and colleagues claim that the IHR “provides a global surveillance infrastructure and orchestrates an appropriate public health response” [2]. This assertion, though, completely misrepresents the nature and reality of the Regulations. The IHR do not provide surveillance infrastructure—as identified above, they are merely a set of guidelines that rely on goodwill to steer inter-state behaviour. In fact, the IHR draw on several existing surveillance networks to accomplish their objective. There is no separate infrastructure. There are few IHR-dedicated staff. Even within Member States, those tasked with responsibility for liaising with the WHO via National IHR Focal Points usually have multiple responsibilities. It is in this regard that invoking the IHR as a way to compel Member States to report cases of AMR will not resolve the “patchy” surveillance, nor address the “financial and technical constraints in large parts of the world” [2]. Improved surveillance is needed, but this will require political leadership and can be accomplished more appropriately through other mechanisms, such as the Global Outbreak Alert and Response Network. The IHR are not the appropriate mechanism to accomplish this work.

## Abbreviations

AMR: antimicrobial resistance
IHR: International Health Regulations
PHEIC: public health emergency of international concern
WHO: World Health Organization

## References

[1] WHO (2011). Antimicrobial resistance.. http://www.who.int/mediacentre/factsheets/fs194/en/index.html.

[2] Harbarth S, Wernli D, Haustein T, Conly J, Carmeli Y, Kickbusch I (2011). A call for action: the application of the International Health Regulations
to the global threat of antimicrobial resistance.. PLoS Med.

[3] WHO (1981). International Health Regulations (1969). Third annotated edition.

[4] WHO (2008). International Health Regulations (2005). Second edition.

[5] Fidler D (2005). From international sanitary conventions to global health security: the new
International Health Regulations.. Chinese Journal of International Law.

[6] Faid M, Gleicher D (2011). WHO Executive Board discusses role and future financing of
WHO.. Health Diplomacy Monitor.

